# The Role of Illness Perception, Hope, and Perceived Social Support in the Prediction of Loneliness in Patients With Acute Coronary Syndrome: A Cross‐Sectional Study

**DOI:** 10.1002/hsr2.71924

**Published:** 2026-05-28

**Authors:** Banafsheh Tehranineshat, Ameneh Faraghi, Nilofar Pasyar, Masoume Rambod

**Affiliations:** ^1^ Department of Nursing, Faculty of Nursing and Midwifery Hormozgan University of Medical Sciences, Bandar Abbas, Iran, and Community Based Psychiatric Care Research Center, Shiraz University of Medical Sciences Shiraz Iran; ^2^ Student Research Committee, School of Nursing and Midwifery Shiraz University of Medical Sciences Shiraz Iran; ^3^ Community Based Psychiatric Care Research Center School of Nursing and Midwifery, Shiraz University of Medical Sciences Shiraz Iran

## Abstract

**Background and Aims:**

Loneliness is a well‐known factor associated with increased mortality risk in cardiovascular patients, and hospitalization in Coronary Care Units (CCU) can exacerbate feelings of loneliness. This study aimed to examine the predictive role of illness perception, hope, and perceived social support on loneliness among patients with Acute Coronary Syndrome (ACS).

**Methods:**

In this descriptive cross‐sectional study, 213 ACS patients admitted to CCUs of university hospitals in southern Iran were enrolled through convenience sampling from October 2021 to March 2022. Data were collected using a demographic characteristics form, illness perception scale, Snyder's Hope Scale, perceived social support questionnaire, and loneliness Scale. Data analysis involved Pearson correlation coefficients and hierarchical linear regression.

**Results:**

Loneliness was most strongly associated with perceived social support (r = − 0.717, *p* < 0.001, r² = 0.52), followed by illness perception (r = 0.591, *p* < 0.001, r² = 0.35) and hope (r = − 0.546, *p* < 0.001, r² = 0.30). Hierarchical regression analysis indicated that illness perception (Step 2: ΔR² = 0.332, β = 0.58, *p* < 0.001, f² = 0.50) and hope (Step 3: β = − 0.32, *p* < 0.001, f² = 0.13) were significant predictors; in the final model, perceived social support emerged as the strongest predictor (β = − 0.52, *p* < 0.001), with the model explaining 56.5% of the variance in loneliness (R² = 0.565).

**Conclusion:**

This study underscores the pivotal role of illness perception and perceived social support in shaping loneliness among ACS patients. Targeted interventions to improve illness perception and enhance social support systems may effectively alleviate loneliness, potentially improving clinical outcomes in this vulnerable population.

## 1 Introduction

Despite the availability of advanced diagnostic tools and therapeutic approaches, cardiovascular disorders are still responsible for disability and death around the world [1]. Cardiovascular diseases are the leading cause of about one‐third of deaths worldwide [2]. The most common type in adults is acute coronary syndrome (ACS). These diseases are responsible for 20%–23% of the disease burden and 46% of total deaths in Iranian population [3].

Because of disease symptoms, ACS patients are admitted to coronary care units (CCUs). The separation from home, unfamiliarity with hospital environment, isolated rooms, limited interactions with family, restricted social contacts, and life‐saving equipment may result in loneliness [4]. As an uncomfortable emotional state, loneliness results from the belief that the social needs of the affected individuals are not fulfilled adequately, either in terms of quantity or, more noticeably, the quality of interpersonal interactions [5, 6]. Based on the conducted studies, cognitive, psychological, and physical functioning is negatively affected by loneliness [2, 6, 7]. Nonetheless, the conducted studies in literature are mostly focused on elderly or community samples, with few hospital‐based investigations [8]. By disrupting social environments, hospitalization leads to or worsens loneliness [9]. As a result, it is critical to identify loneliness factors in early stages to determine clinical interventions for hospitalized individuals.

### 1.1 Literature Review

Illness perception is one of the variables potentially related to loneliness [10]. In accordance with the findings obtained in investigations, low illness perception is related to adverse psychological outcomes, e.g., anxiety, depression, and stress, in individuals experiencing a myocardial infarction [11, 12, 13].

As a concept rooted in positive psychology, hope is closely related to physical health and psychological well‐being outcomes [14]. In accordance with Yildirim et al. (2025), decreased hope among cardiac patients admitted to the Intensive Care Unit (ICU) was significantly correlated with higher loneliness and anxiety [13].

Social support is another factor affecting mental health, which can decrease loneliness. Social support indicates interpersonal exchanges in social networks and provides trust, belief, love, respect, approval, and interest from other individuals [15]. In accordance with recent studies, improved illness perception and perceived social support are directly related to enhanced psychological well‐being and treatment adherence in individuals with cardiovascular diseases [16, 17].

Accordingly, even though loneliness is clinically important‐ and is related to adverse outcomes, e.g., higher anxiety, depression, lengthened hospital stay, and worsened cardiac outcomes, ‐only a few investigations have been specifically focused on such a variable in individuals admitted to the CCU. The CCU‐related features, for example, being a highly stressful environment, restricted family communication, continuous monitoring, severely reduced autonomy of patients, and heavy reliance on specialized care, may potentially increase the levels of stress, loneliness, and psychosocial needs among those individuals [4, 18]. From another viewpoint, restricted direct interaction between patients and their families in the CCU in the communities characterized by strong familial and cultural structures, for example, those in Middle Eastern nations, can result in adverse outcomes, such as higher feelings of anxiety and worry in patients and their families [12]. Nonetheless, scarce evidence is available to comprehensively study loneliness and its influencing factors in the cardiac critical care conditions [19].

A number of studies have shown that illness perception and its aspects—for example, treatment control, emotional representation, and personal control‐may anticipate psychological consequences, including depression and anxiety, among the individuals with coronary artery disease [11, 20]. As a protective construct, hope is related to enhanced life quality and decreased psychological distress, while fulfilling its alleviating function against loneliness feelings [6, 21]. As one of the key factors associated with loneliness perception, social support may result in reduced cardiac distress [13, 15]. Nonetheless, the combined role of the same three critical factors has not been investigated so far for the patients with acute coronary syndrome hospitalized in the CCU. As a result, the lack of a comprehensive investigation that integrally studies hope, social support, and illness perception as loneliness predictors in the CCU indicates a noticeable gap in the scientific literature. By conducting this research, deeper insights are provided into the predictors of loneliness in these patients to pave the way for designing evidence‐based, targeted interventions for hospitalized cardiac patients.

### 1.2 Theoretical Framework

The Biopsychosocial Model was used as the theoretical foundation of the present research. Based on the Biopsychosocial Model, the experience of chronic illnesses, for example, coronary artery syndrome, is caused by the complicated interactions between psychological, social, and biological factors, and thus, the outcomes, including loneliness, are simultaneously affected by the same factors [22, 23]. As a cognitive construct, illness perception may shape the perception of patients regarding to coping resources, and negative illness interpretations can cooccur with reduced hope and diminished perceived social support [24]. Ultimately, these processes may pave the way for the exacerbation or emergence of feelings of loneliness. In addition, given earlier evidence, one may hypothesize that social support, as a protective social factor, and hope, as a positive psychological resource, decrease the feelings of loneliness [13, 15]. Accordingly, this investigation, which is based on the biopsychosocial model, aimed to study the roles of hope, perceived social support, and illness perception in the prediction of loneliness among patients with ACS.

The specific objectives presented below were formulated in the present research:

1. Examining the differences in loneliness levels on the basis of socio‐demographic features among patients with ACS.

2. Investigation of the correlations among hope, illness perception, loneliness, and perceived social support in patients with ACS.

3. Determining the predictors of loneliness‐ including hope, illness perception, demographic characteristics, and perceived social support‐by employing hierarchical linear regression analysis in patients with ACS.

## 2 Methods

### 2.1 Study Design, Setting, and Participants

This descriptive cross‐sectional multicenter investigation was conducted during the October 2021–March 2022 period in three educational hospitals (Shahid Faghihi, Ghalb Alzahra, and Nemaze) affiliated with the Shiraz University of Medical Sciences, Fars Province, Southern Iran. The present research was carried out on the basis of the strengthening of the reporting of observational investigations in epidemiology statement (STROBE), a checklist used for observational research. The study population included the individuals diagnosed by a cardiology specialist with ACS, admitted to the CCU of the three hospitals for nursing treatment and care.

Inclusion criteria included hospitalization in CCU, age range of 18 to 60 years, willingness to participate in the research, ability to read, speak, and write in Persian, and absence of chronic conditions, for example, cancer, advanced cardiovascular diseases (for instance, heart failure), acute pulmonary edema, and rheumatic diseases.

Exclusion criteria were patients experiencing significant emotional crises, for example, divorce within the past 6 months or the death of a close family member, suffering from sensory‐neurological impairments (for instance, vision or hearing loss), cognitive disorders, for example, dementia, known mental health/psychiatric disorders, and the individuals currently using neuropsychiatric medications.

### 2.2 Data Collection

#### 2.2.1 Sampling

In accordance with the study conducted by Pehlivan et al. (2012), which reported a correlation of r = −0.213 (small‐to‐moderate effect size) between loneliness and perceived social support in clinical patients, the minimum sample size was determined [25, 26]. The G*Power software was used with α = 0.05 (two‐tailed), power = 90% (β = 0.10), and Fisher z‐transformation for the purpose of correlation analysis, and the required sample size was determined to be 170. In order to improve robustness and account for ~20% attrition, the final sample included 213 ACS patients, who were admitted to CCUs of teaching hospitals, providing > 90% power for the detection of observed effect sizes.

Also, by visiting the CCUs, the second author (AF) received approval from attending physicians and invited the qualified patients to complete questionnaires voluntarily. In order to ensure comprehension and clarify the questionnaire items, AF remained present there to facilitate a convenience sampling technique in the target population. The following formula was used to calculate the sample size:

#### 2.2.2 Instruments

The tools employed in the present study included Snyder's Hope Scale, the Illness Perception Questionnaire (B‐IPQ) suggested by Brad Bennett et al. Russell's University of California, Los Angeles (UCLA) Loneliness Scale, and the Personal Resource Questionnaire presented by Brandt and Weinert.

A demographic survey was conducted to collect information regarding the gender, age, marital status, education level, income, occupation, place of residence, and the hospitalization site of participants.

Broadbent, Petrie, Mein, and Weinman (2006) developed and validated a 9‐item instrument called the Brief Illness Perception Questionnaire (B‐IPQ) to evaluate the cognitive and emotional perceptions of patients regarding their illness [27]. The first eight items of the questionnaire are scored on a 0–10 scale, and the score interpretation is as follows: 0–1 none or very low, 2–3 low, 4–6 moderate, 7–8 severe, and 9–10 very severe perception levels. Since this study did not focus on disease causation, the ninth item was removed. The three illness perception levels used to categorize the total scores included: high (60–80), moderate (20–60), and poor (0–20). The questionnaire showed good internal consistency (Cronbach's alpha = 0.82), consistent with previous Iranian studies [27, 28]. In this research, the utilized questionnaire showed good internal consistency, with a Cronbach's alpha = 0.82.

The Hope Scale was developed and evaluated psychometrically by Snyder et al. (1991). It is a self‐assessment scale including 12 items distributed across two subscales: Pathways and Agency Thinking [29]. Participants' agreement level for each statement is indicated by a 5‐point Likert scale from “completely agree” to “completely disagree.” The total score ranges between 12 and 60. The total score is categorized as follows: 12–24 low hope, 25–36 moderate hope, and > 36 high hope. The overall reliability of the scale was reported by Nooripour et al. (2022) as Cronbach's alpha = 0.78, with subscale reliabilities of 0.68 for Pathway and 0.73 for Agency Thinking [30]. Cronbach's alpha for the scale in this investigation was 0.84, which reflects good internal consistency.

Developed and evaluated psychometrically by Brandt and Weinert (1987), the Personal Resources Questionnaire aims to measure perceived social support and includes 25 items rated on a 7‐point Likert scale, ranging between 1 (completely disagree) and 7 (completely agree). Total scores are categorized as follows: 25–75 low perceived social support, 76–125 moderate perceived social support, and 126–175 high perceived social support. The Cronbach's alpha reported by Brandt and Weinert for the instrument was 0.89 [31]. By evaluating the content validity index, the validity of the questionnaire was confirmed. The face validity of the scale in Iran was established in 2009, and its reliability was verified with a test–retest coefficient of 0.85 [32]. In this research, a Cronbach's alpha of 0.88 indicated the good internal consistency of the scale.

Developed and evaluated psychometrically by Russell, Peplau, and Cutrona (1980), the UCLA Loneliness Scale includes 20 items; 10 positively and 10 negatively worded statements. Every single item is rated on a 4‐point Likert scale ranging from 1 (never) to 4 (always). Total scores range between 20 and 80, with a mean score of 50. While scores exceeding the mean represent higher loneliness levels, scores below the mean indicate milder loneliness. The scale validity was supported by face validity evaluation, and its test–retest reliability coefficient was reported at 0.89 [33]. By standardizing this scale for a sample of 300 adults, the analysis of confirmatory factor by Zarei et al. (2016) showed good model fit, and the scale indicated robust internal consistency, featuring a Cronbach's alpha of 0.91 [34]. The UCLA Loneliness Scale showed good reliability in this investigation with a Cronbach's alpha of 0.85.

#### 2.2.3 Data Analysis

Data analysis was carried out by employing IBM SPSS Statistics version 22.0 (IBM Corp., Armonk, NY, USA). Descriptive statistics, such as standard deviation, mean, percentage, and frequency, were determined for all variables. In order to evaluate the normality of distributions, the Kolmogorov‐Smirnov test (*p* > 0.05) was used and kurtosis and skewness statistics (all |z | < 1.96) were examined. Pearson's correlation coefficient was used to examine the relationships between continuous variables. Correlation strength followed Cohen's conventions: |r | = 0.50–1.00 (large), 0.30–0.50 (medium), 0.10–0.30 (small), 0.00–0.10 (negligible) [26].

Hierarchical multiple linear regression was carried out in order to determine the predictors of loneliness. In this technique, demographic variables (gender, age, marital status, income, and education level) were entered in the first step. This was followed by entering hope, perceived social support, and illness perception in the next steps. Normality and linearity assumptions were checked using Kolmogorov–Smirnov tests, probability plots, and residual analyzes. Multicollinearity was analyzed by the variance inflation factor (VIF), with all < 10 values reflecting insignificant multicollinearity. Independence of errors was validated using the Durbin‐Watson statistic (1.5–2.5 acceptable). Mediation analysis utilized the PROCESS macro (Model 4, 5000 bootstraps) of Hayes with bias‐corrected confidence intervals. The tests were totally two‐tailed (α = 0.05) [35, 36, 37]. Statistical terms are defined as follows: β, standardized regression coefficient; R², proportion of variance explained; ΔR², additional variance explained in each hierarchical step; CI, 95% confidence interval; F, F‐statistic; η²p, partial eta squared; VIF, variance inflation factor; P, two‐tailed probability value.

## 3 Results

The study population included 213 ACS patients. Table 1 shows their demographic specifications. Most of the subjects were married individuals (90.6%) and male patients (77%). The mean age of the subjects was 51.73 years (SD = 7.60). The largest proportion was from the 50–59 age group (52.6%). The majority of participants were employed (53.5%), and the most frequent educational level was high school diploma (64.8%).

**Table 1:** The demographic characteristics of the participants in this study.

Variables	*N*	%	Feeling of loneliness mean (SD)	CI 0.95%	Test, *p*‐value
Age					
< 39	14	6.6	36.28 ± 12.23	29.22–43.34	
40–49	55	25.8	32.55 ± 8.04	30.36–34.75	F = 0.66, *p* = 0.57 *
50–59	112	52.6	34.06 ± 9.88	32.21–35.91	
> 60	32	15.0	33.31 ± 9.55	29.86–36.75	
Gender					
Female	49	23.0	33.41 ± 8.86	30.84 ± 35.99	t = 0.23, *p* = 0.80 **
Male	164	77.0	33.79 ± 9.76	32.29 ± 35.30	
Education					
High school	138	64.8	33.93 ± 9.10	32.39–35.47	
Diploma	53	24.9	33.60 ± 10.57	30.67–36.51	F = 1.09, *p* = 0.40*
Associate Degree	11	5.2	35.63 ± 12.17	27.45–43.81	
Bachelor	5	2.3	25.8 ± 5.06	19.50–32.09	
Masters	6	2.8	32.66 ± 6.28	26.07–39.25	
Marital status					
Single	9	4.2	37.66 ± 10.51	29.58–45.74	
Married	193	90.6	33.20 ± 9.12	31.90–34.50	F = 2.09, *p* = 0.10*
Divorced	3	1.4	37 ± 10.81	10.12–63.87	
Widow	8	3.8	40.25 ± 15.35	27.40–53.09	
Job					
Employed	114	53.5	33.73 ± 9.69	31.93–35.53	
Unemployed	32	15.0	35.31 ± 9.53	31.87 ± 38.75	F = 0.53, *p* = 0.65**
Retired	21	9.9	32.04 ± 10.07	27.46–36.63	
Housewife	46	21.6	33.28 ± 9.06	30.56–36.01	
Income/Rials					
< 50 million	22	10.3	40.18 ± 9.20	36.10–44.26	
50–100 million	94	44.1	33.19 ± 8.75	31.39–34.98	F = 4.63, *p* = 0.004*
100–150 million	61	28.6	33.76 ± 9.90	31.20–36.32	
> 150 million	36	16.9	31.02 ± 9.75	27.72–34.32	
Hospital					
Nemazee	15	7.0	33.73 ± 13.17	26.43–41.03	
Shahid Faghihi	25	11.7	37.32 ± 10.93	32.80–41.83	F = 2.06, *p* = 0.12*
Ghalb Alzahra	173	81.2	33.18 ± 8.90	31.84–34.52	
Place of living					
Urban	173	81.2	34.11 ± 9.66	32.65–35.56	t = 1.26, *p* = 0.20**
Rural	40	18.8	32 ± 8.95	29.13–34.86	

Abbreviation: CI, confidence interval.

* ANOVA.

** *t*‐test.

Descriptive statistics of illness perception, hope, perceived social support, and loneliness in patients with ACS are presented in Table 2.

**Table 2:** The mean scores of illness perception, hope, perceived social support, and loneliness in ACS patients.

Variable	Mean ± SD (total score)	Observed range	Highest‐scoring item (Mean ± SD)	Lowest‐scoring item (Mean ± SD)
Illness perception	44.46 ± 15.21	7–76	Worry about illness (6.62 ± 2.87)	Belief in treatment effectiveness (2.17 ± 1.94)
Hope	40.00 ± 6.31	23–55	Finding multiple ways to achieve life goals (3.88 ± 0.88)	Worrying about problems (2.07 ± 0.88)
Perceived social support	135.81 ± 17.60	71–168	Enjoyment in making others happy (6.62 ± 0.80)	Inability to rely on relatives and friends (4.15 ± 1.85)
Loneliness	33.71 ± 9.54	20–64	Having common interests with others (2.08 ± 0.69)	Meaninglessness of relationships (1.40 ± 0.73)

### 3.1 Associations Between Demographic Characteristics and Loneliness Among ACS Patients

Based on the analysis of variance (ANOVA), only income was significantly related to loneliness (ANOVA: F (3,209) = 4.63, η_p² = 0.06 [small effect], *p* = 0.004). No significant relationship was observed for other demographic variables (all *p* > 0.05) (see Table 1). No significant correlation was observed between loneliness and age (Pearson's r = 0.01 [negligible effect], 95% CI [–0.10, 0.12], *p* = 0.57).

### 3.2 The Correlation Between Loneliness and Illness Perception, Hope, and Perceived Social Support in the Patients With ACS

According to the Pearson correlation analysis, significant negative correlations were found between hope (r = ‐0.546 [large effect], 95% CI [–0.62, –0.46], *p* < 0.001) and perceived social support (r = –0.717 [large effect], 95% CI [–0.77, –0.65], *p* < 0.001) and loneliness. A significant positive correlation was observed between illness perception and loneliness (r = 0.591 [large effect], 95% CI [0.51, 0.66], *p* < 0.001) (see Table 3).

**Table 3:** The correlation between hope, perceived social support, illness perception, and loneliness in the Acute coronary syndrome patients.

Variables	Illness perception	Hope	Perceived social support	Feeling of loneliness
Illness perception	1			
Hope	–0.53**	1		
Perceived social support	–0.0594**	0.658**	1	
Feeling of loneliness	0.591**	–0.546**	–0.717**	1

*Note:* Values are Pearson correlation coefficients (*n* = 213).

** *p* < 0.01 (two‐tailed).

### 3.3 Predictors of Loneliness in ACS Patients: A Hierarchical Linear Regression Analysis

Table 4 shows that a hierarchical linear regression analysis was carried out in order to analyze the predictors of loneliness among ACS patients. Furthermore, as Table 5 indicates, given that hope, illness perception, perceived social support, and loneliness Kurtosis and Skewness z‐scores ranged from −1.96 to 1.96, all variables featured a normal distribution. In addition, Kolmogorov‐Smirnov tests indicated normality for regression residuals (*p* = 0.28) and all variables (*p* > 0.05), which satisfies parametric assumptions. Given that the value of Durbin‐Watson statistic for the regression model was 2.09, it falls within the commonly accepted range of 1.5–2.5. Such a result indicates that the autocorrelation among the residuals was insignificant. As a result, the assumptions of normality and independence of errors were sufficiently approved for the purpose of regression analysis. Given that Variance Inflation Factor (VIF) was < 10 for all variables, the collinearity diagnostics showed no multicollinearity among independent variables (Table 4).

**Table 4:** Hierarchical linear regression analysis used to determine the predictors of loneliness in patients with acute coronary syndrome.

Model		Standardized coefficients									Collinearity statistics	
		Beta	t	*p*‐ value	95.0% Confidence interval for B	R	R^2^	ΔR^2^		F change, *p*‐value	Tolerance	VIF
1	(Constant)		6.21	< 0.001	25.46–49.11	0.19^a^	0.037	0.03		1.99, 0.09		
	Age	–0.01	–0.14	0.88	–0.18 to 0.16						0.958	1.044
	Gender	0.04	0.64	0.52	–2.17 to 4.26						0.911	1.097
	Education	0.03	0.42	0.67	–1.35 to 2.10						0.663	1.509
	Marital status	0.38	0.79	0.21	–1.18 to 3.26						0.9	1.10
	Income	–0.21	–2.60	0.01	–3.95 to –0.54						0.715	1.399
2	(Constant)		3.39	< 0.001	7.44 to 28.02	0.60^b^	0.36	0.332		107.29, < 0.001		
	Age	–0.01	–0.22	0.82	–0.15 to 0.12						0.958	1.044
	Gender	0.07	1.31	0.18	–0.86 to 4.36						0.909	1.100
	Education	0.03	0.43	0.66	–1.09 to –1.71						0.663	1.509
	Marital status	0.41	1.38	0.08	–1.25 to 2.12						0.89	1.11
	Income	–0.12	–1.83	0.06	–2.68 to 0.09						0.702	1.424
	Illness perception	0.58	10.35	< 0.001	0.29–0.43						0.972	1.028
3	(Constant)		6.16	< 0.001	29.15–56.57	0.66^c^	0.44	0.074		26.18, < 0.001		
	Age	–0.04	–0.77	0.44	–0.18 to 0.08						0.947	1.055
	Gender	0.06	1.18	0.23	–0.99 to 3.94						0.907	1.102
	Education	0.05	0.84	0.40	–0.76 to 1.89						0.659	1.517
	Marital status	0.85	1.39	1.66	–0.77 to 4.47						0.88	1.13
	Income	–0.08	–1.39	0.16	–2.26 to 0.38						0.695	1.439
	Illness perception	0.41	6.70	< 0.001	0.18–0.33						0.708	1.411
	Hope	–0.32	–5.11	< 0.001	–0.681 to –0.30						0.682	1.466
4	(Constant)		9.79	< 0.001	55.26–83.13	0.75 ^d^	0.56	0.124	57.41, < 0.001			
	Age	0.01	0.22	0.82	–0.10 to 0.13						0.928	1.078
	Gender	0.003	0.06	0.94	–2.14 to 2.28						0.883	1.133
	Education	0.05	0.92	0.35	–0.62 to 1.72						0.659	1.517
	Marital status	0.47	0.40	0.68	–1.84 to 2.7						0.86	1.16
	Income	–0.06	–1.19	0.23	–1.87 to 0.46						0.693	1.443
	Illness perception	0.24	4.02	< 0.001	0.07 to 0.22						0.601	1.665
	Hope	–0.07	–1.07	0.29	–0.30 to 0.08						0.502	1.992
	Perceived social support	–0.52	–7.57	< 0.001	–0.35 to 0.20						0.455	2.199

**Table 5:** Normality testing for illness perception, hope, perceived social support, and loneliness.

Variables	Skewness	Std. error	Skewness z‐scores	Kurtosis	Std. error	Kurtosis z‐scores
Illness perception	–0.04	0.16	–0.25	–0.45	0.33	–1.36
Hope	–0.27	0.16	–1.68	0.03	0.33	0.09
Perceived social support	–0.67	1.68	–0.39	0.53	0.33	1.60
loneliness	0.28	0.16	1.75	–0.05	0.33	–0.15

In step 1, demographic variables explained 3.7% of loneliness variance (R² = 0.037 [small effect], F (5, 207) = 1.99, *p* = 0.096) with income as a significant parameter (β = −0.21, 95% CI [**–**3.95, **–**0.54], *p* = 0.01; Table 4). In accordance with the earlier research and theoretical foundations [23, 38, 39, 40], gender, age, marital status, and education were entered in the first block of the hierarchical regression model as control variables. Nonetheless, in this sample, such variables had insignificant relationship with the dependent variable.

Illness perception addition explained ΔR² = 0.332 [large effect] additional variance (F_change (1, 206) = 107.29, *p* < 0.001) in step 2. According to the results, illness perception (β = 0.58, 95% CI [0.29, 0.43], *p* < 0.001) was still the significant predictor of loneliness. Nonetheless, gender (β = 0.07, *p* = 0.18), age (β = −0.01, *p* = 0.82), marital status (β = 0.41, *p* = 0.08), income (β = −0.12, *p* = 0.06), and education (β = 0.03, *p* = 0.66) were insignificant predictors in this step.

In step 3, the addition of hope could explain ΔR² = 0.074 [medium effect] (F_change (1, 205) = 26.18, *p* < 0.001). Both hope (β = −0.32, 95% CI [−0.68, −0.30], *p* < 0.001) and illness perception (β = 0.41, 95% CI [0.18, 0.33], *p* < 0.001) predicted loneliness significantly. Nonetheless, gender (β = 0.06, *p* = 0.23), age (β = −0.04, *p* = 0.44), marital status (β = 0.85, *p* > 0.99), income (β = −0.08, *p* = 0.16), and education (β = 0.05, *p* = 0.4) were insignificant predictors in this step.

In step 4, perceived social support was capable of explaining ΔR² = 0.124 [medium effect] (F_change (1, 204) = 57.41, *p* < 0.001). In the final model, perceived social support (β = −0.52, 95% CI [−0.35, **−**0.20], *p* < 0.001) and illness perception (β = 0.24, 95% CI [0.07, 0.22], *p* < 0.001) served as significant predictors. The full model was capable of explaining 56.5% of the variance in loneliness (R² = 0.565) (Table 4).

The mediation analysis carried out by employing Hayes' PROCESS macro (Model 4) evaluated the contribution of illness perception to patients' feeling of loneliness mediated by perceived social support. In total, illness perception significantly affected the subjects' loneliness (B = 0.3703, SE = 0.0349, t = 10.61, *p* < 0.001). Although the direct impact of illness perception on loneliness remained significant when controlling for perceived social support, its magnitude declined (B = 0.1598, SE = 0.0360, t = 4.44, *p* < 0.001). This indicates a partial mediation. In addition, the indirect contribution of illness perception to loneliness through perceived social support was significant (effect = 0.2105, BootSE = 0.0315), with a 95% bootstrap confidence interval of (0.1517, 0.2748) that excluded zero. Such a fact suggests that perceived social support partially mediates the association between loneliness and illness perception.

## 4 Discussion

This research discovered that among demographic variables, only income showed a significant association with loneliness in patients with ACS, which supports the evidence that socioeconomic benefit is associated with a higher level of loneliness and poorer outcomes regarding the mental health [39]. Financial strain can result in limited accessibility of supportive resources and intensified emotional vulnerability in the course of hospitalization. Contrarily, loneliness was insignificantly related to gender, age, education, residence, or occupation. This result is contrary to community‐based investigations, which reported higher loneliness among females, older adults, and the people with lower educational levels [23, 38, 40]. Such disagreements may indicate the acute nature of cardiac illness, whereby the stressors associated with illness outweigh typical demographic impacts [41]. The significant effect of income underlines the significance of considering financial hardship when evaluating psychosocial demands and integrating socioeconomic support into comprehensive cardiac care.

Based on the results of this investigation, illness perception among patients with ACS was moderate and positively related to loneliness. This is in agreement with earlier studies claiming that negative illness perceptions are associated with poorer psychological outcomes, for example, depression, social withdrawal, and anxiety [42, 43]. Similar findings obtained for the patients suffering from ischemic heart disease indicate that maladaptive illness perceptions can result in undermined engagement in health‐promoting behaviors as well [44]. From a psychological viewpoint, more adaptive illness perceptions may result in improved coping, reduced emotional distress, the promotion of a sense of social connectedness, and consequently, mitigated loneliness [45]. Thus, interventions aimed at illness perception, alongside the improvement of social support and hope, can enhance the life quality and psychological well‐being of patients with ACS.

Based on the results of this research, hope levels were moderate to high and inversely related to loneliness among patients with ACS. This indicates the protective role of hope against social isolation. Such a finding is in line with earlier studies showing that hope leads to the promotion of psychological well‐being among cardiac and other patient populations [46, 47, 48]. Once motivation, engagement in adaptive coping behaviors, and emotional resilience are enhanced, hope can overcome the adverse psychological impacts of acute illness. As a result, the integration of hope‐improving strategies into patient care can help reduce loneliness and enhance quality of life and psychological adaptation among patients with ACS.

Based on the findings of this study, patients with ACS showed higher levels of perceived social support. This was inversely correlated with loneliness, which is in agreement with the findings of earlier studies associating social support with enhanced psychological well‐being [49, 50]. Contrarily, a number of investigations have shown lower levels of perceived support among the individuals with cardiovascular conditions, which indicates potential variability as a result of social, clinical, or cultural settings [51, 52]. Perceived social support presents informational, practical, and emotional resources facilitating the strategies to cope with acute illness, promoting psychological adaptation, and reducing social isolation. The high support levels witnessed in the present investigation may indicate robust family involvement, which highlights the significance of strengthening and evaluating social support as part of comprehensive care strategies recommended for patients with ACS.

In accordance with the final hierarchical regression model, the only significant predictors of loneliness in patients with ACS were perceived social support and illness perception. In addition, mediation analysis showed that perceived social support could partially mediate the relationship between loneliness and illness perception. This indicates that more adaptive illness perceptions lead to decreased loneliness partly via improved social support. These findings are in line with earlier findings underscoring the combined role of social and cognitive resources in the promotion of psychological adjustment and reduction of social isolation [42, 53]. As a result, interventions for ACS patients should be focused not only on the improvement of illness‐related understanding but also on the strengthened networks of social support so as to effectively promote psychological well‐being and alleviate loneliness.

### 4.1 Limitation

A number of limitations on the present study are as follows. First, the use of convenience sampling may have resulted in selection bias. This is because the subjects were recruited on the basis of accessibility instead of random selection. This could lead to reduced sample representativeness and limited generalizability of the findings to a broader community of patients suffering from ACS. Second, this research was exclusively concentrated on patients with ACS. As a result, the applicability of the results to other patient populations with acute/chronic conditions may be subject to limitations. Third, data collection was conducted by employing self‐reported measures, and these data may be subject to potential biases, for example, social desirability and recall bias.

### 4.2 Clinical Implications

Patients should be educated by the healthcare providers to develop accurate, positive illness perceptions and strengthen informational, practical, and emotional support from peers, clinical teams, and family. The enhancement of social support may lead to decreased loneliness, enhanced psychological well‐being, and potentially enhanced clinical outcomes in ACS patients.

## 5 Conclusion

Robust support networks and the promotion of adaptive illness perceptions for the ACS patients lead to significant reduction in loneliness feeling and improved psychological well‐being. Interventions should be aimed at educating patients about their condition and reinforcing support systems. Future studies should be focused on exploring longitudinal effects and other psychosocial factors in order to guide tailored, effective supportive care solutions.

## Author Contributions

**Banafsheh Tehranineshat:** conceptualization, investigation, formal analysis, writing—review and editing. **Ameneh Faraghi:** investigation, data curation, project administration, software and writing—original draft preparation. **Nilofar Pasyar:** conceptualization, supervision, methodology, writing—review and editing. **Masoume Rambod:** conceptualization, supervision, writing—review and editing.

## Ethics Statement

Written informed consent was obtained from all participants. The study was conducted in accordance with the revised Declaration of Helsinki. Anonymity and confidentiality were ensured for all participants. Ethical approval was granted by the Institutional Research Ethics Committee of Shiraz University of Medical Sciences, Shiraz, Iran (Ethics code: Ir.SUMS.REC.1400.042).

## Conflicts of Interest

The authors declare no conflicts of interest.

## Transparency Statement

The lead author Nilofar Pasyar affirms that this article is an honest, accurate, and transparent account of the study being reported; that no important aspects of the study have been omitted; and that any discrepancies from the study as planned (and, if relevant, registered) have been explained.

## Data Availability

The data that support the findings of this study are available from the corresponding author upon reasonable request, in accordance with ethical and privacy considerations.
